# Climate-driven variation in the phenology of juvenile Ixodes pacificus on lizard hosts

**DOI:** 10.21203/rs.3.rs-5671938/v1

**Published:** 2024-12-23

**Authors:** Samantha Sambado, Amanda Sparkman, Andrea Swei, Andrew J MacDonald, Hillary S Young, Jordan Salomon, Arielle Crews, Kacie Ring, Stephanie Copeland, Cheryl J Briggs

**Affiliations:** University of California Santa Barbara; Westmont College; San Francisco State University; University of California Santa Barbara; University of California Santa Barbara; Texas A&M University; San Mateo County Mosquito and Vector Control; University of California Santa Barbara; University of California Santa Barbara; University of California Santa Barbara

**Keywords:** Ixodes pacificus, Sceloporus occidentalis, latitudinal gradient, Mediterranean climate, host-attached ticks, phenology

## Abstract

**Background:**

Ectothermic arthropods, like ticks, are sensitive indicators of environmental changes, and their seasonality plays a critical role in tick-borne disease dynamics in a warming world. Juvenile tick phenology, which influences pathogen transmission, may vary across climates, with longer tick seasons in cooler climates potentially amplifying transmission. However, assessing juvenile tick phenology is challenging in climates where desiccation pressures reduce the time ticks spend seeking blood meals. To improve our understanding of juvenile tick seasonality across a latitudinal gradient, we examine *Ixodes pacificus* phenology on lizards, the primary juvenile tick host in California, and explore how climate factors influence phenological patterns.

**Methods:**

Between 2013 and 2022, ticks were removed from 1,527 lizards at 45 locations during peak tick season (March-June). Tick counts were categorized by life stage (larvae and nymphs) and linked with remotely sensed climate data. Juvenile phenology metrics, including abundance, date of peak abundance, and temporal overlap between larval and nymphal populations, were analyzed along a latitudinal gradient, including tick abundances on lizards, Julian date of peak mean abundance. Generalized Additive Models (GAMs) were applied to assess climate-associated variation in juvenile abundance on lizards.

**Results:**

Mean tick abundance per lizard ranged from 0.17 to 47.21 across locations, with the highest in the San Francisco Bay Area and lowest in Los Angeles, where more lizards had zero ticks attached. In the San Francisco Bay Area, peak nymphal abundance occurred 25 days earlier than peak larval abundance. Temporal overlap between larval and nymphal stages at a given location varied regionally, with northern areas showing higher overlap. We found that locations with higher temperatures and increased drought stress were linked to lower tick abundances, though the magnitude of these effects depended on regional location.

**Conclusion:**

Our study, which compiled 10 years of data, reveals significant regional variation in juvenile *I. pacificus* phenology across California, including differences in the abundance, peak timing, and temporal overlap. These findings highlight the influence of local climate on tick seasonality, with implications for tick-borne disease dynamics in a changing climate.

## Background

Pathogen vectors, such as ticks, pose serious risks to the health of both humans and animals (1). Ticks, as ectotherms, are highly sensitive to environmental conditions, and their population dynamics – including abundance, seasonality, and host associations - are significantly affected by weather and climate conditions (2–4). Notably, juvenile ticks, especially larval and nymphal *Ixodes* species, exhibit seasonal activity patterns that directly impact local pathogen prevalence of non-vertically transmitted pathogens such as *Borrelia burgdorferi*, the causative agent of Lyme disease (5–7). For instance, if uninfected larvae emerge prior to infected nymphs, the opportunities for acquiring infectious blood meals diminish (7). Conversely, the emergence of infected nymphs prior to larvae can enhance pathogen transmission by increasing the likelihood of larvae feeding on an infected blood meal and becoming infectious nymphs in subsequent seasons (7).

Lyme disease is a key example of how the seasonality of juvenile ticks can influence the risk to human health. The prevalence of Lyme disease is highly variable across the United States (US) even in areas where environmental conditions favor both competent *Ixodes* ticks and their vertebrate hosts (8–10). Various ecological hypotheses have been proposed to explain these discrepancies, including differences in vertebrate community structures, habitat fragmentation that increases human-tick interactions, and strain-specific variations in the Lyme disease bacterium (11–13). However, many studies focus on small spatial scales when examining the relationship between these factors and disease risk, potentially overlooking the critical influence of larger scale climatic factors across the full range of *Ixodes scapularis* and *Ixodes pacificus*.

Linking climatic conditions to juvenile *Ixodes* seasonality is essential for explaining geographic patterns of Lyme disease risk and implications for how climate change may alter this risk. For instance, juvenile seasonality has been cited as an explanation for heightened Lyme disease incidence in the cooler northeastern US compared to the warmer southeastern regions (3, 14–17). However, substantial time and resource constraints often hinder systematic longitudinal and latitudinal studies (16, 18). Latitudinal studies that have successfully collected juvenile ticks in drier regions have typically relied on sampling juvenile tick hosts, such as lizards, rather than using traditional tick collection methods like dragging or flagging (14, 19, 20). The difficulties from sampling juvenile ticks in drier regions is due to their behavioral responses to desiccation pressures, which lead them to prefer ground-level leaf litter – where desiccation risk is lower and hosts like lizards and rodents exist – over questing in aboveground vegetation (21–24). Although lizards do not directly provide infectious blood meals, they may serve as proxies for broader juvenile tick activity patterns, potentially reflecting rodent tick burden dynamics (25–27).

California’s western coastal region is particularly well-suited for investigating climate effects on juvenile phenology patterns for several reasons. First, Lyme disease risk is disproportionately high in northwestern California, with Mendocino County reporting an incidence rate of 3.9 cases per 100,000 people, compared to the state average of 0.2 (28) (CDPH 2021). Second, *I. pacificus* ticks and their preferred larval/nymphal host, the western fence lizard (*Sceloporus occidentalis*), share a broad latitudinal distribution (10). Third, the region encompasses significant microclimate variability, with coastal areas exhibiting higher humidity levels and lower temperatures than nearby inland areas (19, 29, 30). Finally, areas with significant desiccation stress have historically shown limited success in assessing juvenile tick activity using traditional sampling methods such as flagging or dragging (20, 22, 31). Despite these factors, a systematic statewide analysis focusing on the preferred host of juvenile *I. pacificus* has not been conducted.

Given these factors, this study aims to investigate juvenile phenology patterns across California’s latitudinal gradient in relation to climate variability, addressing key gaps in juvenile tick ecology and providing insight into potential ecological mechanisms driving variations in Lyme disease risk. We address challenges in collecting juvenile ticks in drier regions by incorporating data from lizard surveys and documenting attached juvenile *I. pacificus* across a latitudinal gradient, thereby creating a unique comprehensive dataset. By integrating existing tick research with high-resolution climate data and using Generalized Additive Models (GAMs) (32), we have two main objectives: 1) to characterize seasonal patterns of juvenile tick abundance on their preferred host across latitudes, and 2) explore the relationship between juvenile abundances and climate conditions across California’s climatically diverse regions. Understanding the seasonality of juvenile ticks and their climate-driven variation will enhance Lyme disease risk predictions and inform public health strategies, as well as guide future empirical tick-related research.

## Methods

The data for this study were collected from 2013 to 2022, primarily during the peak juvenile activity months of *I. pacificus* ticks, which are March through June. This aggregated dataset includes 45 unique sampling locations and encompasses a total of 253 sampling days (Additional file 1: Table S1 and Table S2). Of the 45 locations, 93% (n = 42) were sampled multiple times, with 84% (n = 38) being sampled three or more times (Additional file 2: Fig. S1). The data reflect the collective efforts of various lab groups engaged in ecological research, which included both lizard sampling and tick burden assessments (Additional file 2: Fig. S2 and Table S1). The collectors – MacDonald, Sambado, Sparkman, Swei, and Young – led the original study design (19, 20, 29, 33).

### Study area

**Figure 1:**
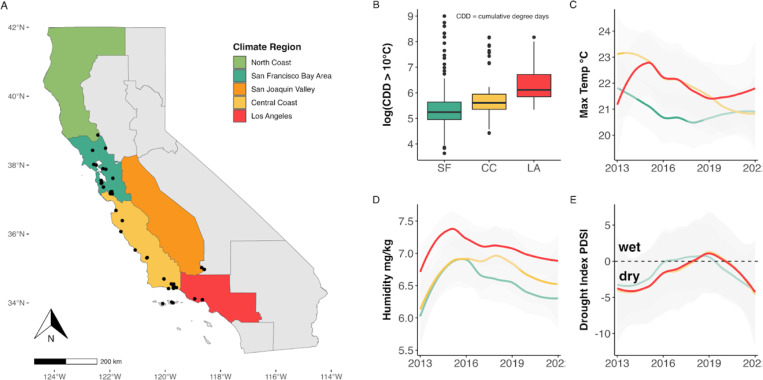
(A) Map of California, color-coded by climate regions. Black dots indicate unique sampling locations where lizard-attached ticks were collected. (B) Box plot showing log_10_ transformed cumulative degree days (CDD) across the most frequently sampled climate regions (SF = San Francisco Bay Area, CC = Central Coast, LA = Los Angeles). (C) Mean monthly maximum temperature. (D) Mean monthly specific humidity. (E) Mean drought index (Palmer Drought Severity Index). Shaded gray area represents 95% confidence intervals.

Sampling was conducted across various locations in California, primarily in the western coastal region. This region has a Mediterranean climate, characterized by relatively wet, cool winters that transition into warm, dry summers (34). Sampling locations spanned a latitudinal gradient from Lake County in the north (39°05’24.00 N, 122°45’36.00 W) to Los Angeles County in the south (34°16’50.96’’ N, 119°17’ 40.56 W) (Fig. 1). These sites were situated in ecosystems known to harbor tick populations, typically including mixed oak woodlands, oak savannas, or coastal chaparral habitats (10, 31). Additional details about the study locations can be found in Additional file 2 and in previously published studies (19, 20, 29, 33). To establish climatically relevant groupings of field sites across our study area, we overlaid location coordinates onto Cal-Adapt’s climate region polygons (North Coast Region, San Francisco Bay Area Region, San Joaquin Valley Region, Central Coast Region, Los Angeles Region) as defined by California’s 4th Climate Change Assessment (35).

### Field collection

Lizards were primarily captured using standard lassoing techniques, which involved a dental floss or fishing line lasso attached to the tip of a fishing pole, following the methodology of Swei et al. (36). In some instances, lizards were captured by hand from under cover boards. Sampling typically occurred midday to target peak basking times. Ticks found on a lizard’s ears and nuchal pouches were carefully removed with fine-tipped forceps and placed in 70% ethanol for later identification in the lab. Detailed methods for each collector can be found in Additional file 3: Text S1.

### Remotely sensed climate data

All climate data in this study were obtained from the gridded meteorological dataset, gridMET (37). This dataset provides daily, high-resolution (~ 4 km) meteorological data across the contiguous US. In line with the tick-climate literature (38–40), the primary climate variables used in our analysis included near-surface specific humidity (sph; kg/kg), maximum temperature (tmmx; °C), and minimum temperature (tmmn; °C). Derived variables included the monthly Palmer Drought Severity Index (PDSI), a standardized index that estimates relative soil moisture conditions based on a simplified soil water balance, calculated every five days. For instance, a PDSI value greater than 4 indicates very wet conditions, while a value less than − 4 signifies extreme drought. For each site location coordinates (n = 45), data were downloaded for the continuous period from 2013 to 2022 to calculate various indices, such as mean maximum and minimum temperature in each season (spring, summer, fall, winter), and mean climate variables for all months. Using maximum and minimum temperatures, we calculated the cumulative degree days (CDD) for each year and location from January 1st to March 31st. This date range was selected to capture spring dynamics before the peak juvenile season, based on the assumption that a warmer spring (i.e., higher CDD) would result in earlier tick activity (41). The base temperature was set at 10°C, a known threshold for the thermal accumulation required by ectothermic invertebrates in California (42). Daily climate variables were matched to the date of lizard-tick sampling. The gridMET data was accessed on October 23, 2024.

### Statistical analyses

In our analysis, we focus on the abundance of ticks collected from individual lizards, which we aggregate to represent the mean tick abundance for each location or climate region per month. We further categorize the tick abundances by life stages – specifically larval and nymphal ticks. Since most sampling locations (n = 41) are concentrated in three regions, we focus our analysis on the San Francisco Bay Area, Central Coast, and Los Angeles regions. However, in supplementary files we include summary statistics for two less frequently sampled but important areas of comparison: the North Coast and San Joaquin Valley regions (Additional file 1, Additional file 2, Additional file 3).

### Characterizing the seasonal patterns of juvenile ticks

Juvenile seasonal patterns are characterized by several metrics that capture various aspects of tick population dynamics including phenology (43, 44). We constrain our analysis to tick abundance data collected between March 1st and June 30th, which coincides with peak juvenile tick activity and corresponds to Julian dates 60 to 181 in non-leap years. During this period, we quantify the following juvenile seasonal patterns for each climate region and life stage: juvenile abundances on lizards, key phenological dates (e.g., date of peak abundance), and temporal overlap metrics. More details on these methods are found below.

Juvenile abundance refers to the number of ticks removed from individual lizards, with the mean and standard deviation calculated per month and within each sampling location. Key phenological dates include the Julian date of peak abundance (i.e., the day of maximum mean abundance for each year), as well as the first and last appearance dates for each life stage. However, due to differences in sampling schedules, the first and last appearance dates serve as general indicators rather than definitive markers for a given region (see Additional file 4: Table S1). Finally, we assess temporal overlap between larval and nymphal populations at each location using two metrics: Kernel Density Estimation (KDE) and the Jaccard similarity coefficient (Jaccard Index). KDE is a non-parametric method used to estimate population density, providing a smooth estimate of overlap between larval and nymphal distributions. The Jaccard Index evaluates the degree of temporal overlap between the two life stages at each location. Together, these metrics provide insights into the temporal dynamics of juvenile tick populations. Higher values for both KDE and Jaccard Index indicate greater overlap between larval and nymphal populations, implying more synchronized activity within a location. For further justification of these methods, see Additional file 3: Text 2.

### Exploring the relationship between juvenile tick abundances and climate

We hypothesize that, as ectotherms, tick burdens are influenced by abiotic factors, particularly monthly temperatures that trigger seasonal behaviors and sufficient humidity to mitigate daily desiccation pressures. Additionally, we aim to capture the long-term effects of local drought conditions on tick populations, if any. We fit Generalized Additive Models (GAMs) to assess the relationship between tick burdens (i.e., tick count per lizard, including lizards with no ticks) and monthly abiotic variables of the sampling month, including maximum temperature (tmmx), near-surface specific humidity (sph), and drought index (PDSI) (32). These variables were standardized for interpretation and checked for multicollinearity (variance inflation factor < 3).

To capture potential nonlinear relationships between tick abundance and climate variables, we used cubic regression spline smoothing terms (bs = “cr”) for continuous predictors and included an interaction term between climate region and predictors to account for geographic variation in climate and tick burdens. To address spatial autocorrelation, we include Gaussian processing smoothing terms (bs = “gp”) for the latitude and longitude of each sampling location. The optimal smoothing parameter for each smooth term was determined using restricted maximum likelihood (REML) estimation (32). The model was specified using a Tweedie distribution and a log link function, which is appropriate for modeling overdispersed, zero-inflated count data like tick burdens (45). Due to limited observations in the North Coast and San Joaquin Valley regions and their relatively close geographic distances from other climate regions, we group those locations with the San Francisco Bay Area and Los Angeles regions, respectively. We ran this model first for juvenile abundances of individual lizards, followed by separate models for larval and nymphal burdens.

To evaluate model performance, we used the *mgcv* package to fit both GAMs and perform model diagnostics (32). Within each model grouping, we compared fit using Akaike’s Information Criterion (AIC), selecting the model with the lowest AIC as the best-fitting model. The significance of each predictor was evaluated using likelihood ratio tests based on the estimated degrees of freedom (edf), and the model fit was compared to a null model with no smooth terms. We visualized the relationship of our outcomes to predictive values using the plot function to produce partial effects plots that show the predictor’s effect when other variables are at their average value. Refer to Additional file 3: Text S2 and Fig. S1 for additional model justifications.

### Software

All statistical analyses were conducted in RStudio version 4.4.1 (46) with a significance level set at p < 0.05. Climate data from gridMET were obtained through the *c* lim *ateR* package (47). Data cleaning and visualizations were conducted with *tverse* and *ggplot*2 packages, respectively (48, 49). To assess multicollinearity among climate variables, we used the vif function from the *car* package (50). The code for figures and analysis is available at: https://github.com/sbsambado/ca_lizardburden.

## Results

### Field collection summary

In total, 1,527 individual lizards were sampled. The majority (62%) of the identified lizards were western fence lizards (*S. occidentalis*), while the remainder included alligator lizards (*Elgaria* spp; 37%) and common side-blotched lizards (*Uta stansburiana*; < 1%). Unidentified lizards were presumed to be *S. occidentalis* based on the objectives of each collecting group. Consequently, we assumed that the majority of attached ticks processed were *I. pacificus*, supported by historical data on tick attachment to *S. occidentalis*, *Elgaria* spp., and *U. stansburiana* (25, 26, 51). Throughout the study period, a total of 9,338 ticks were counted on lizards. However, not all ticks were identified to larval or nymphal stages. Among those identified, 4,197 were larvae and 3,239 were nymphs, representing 80% of the attached juvenile ticks.

### Seasonal patterns of juvenile ticks vary

#### Tick abundances:

**Figure 2:**
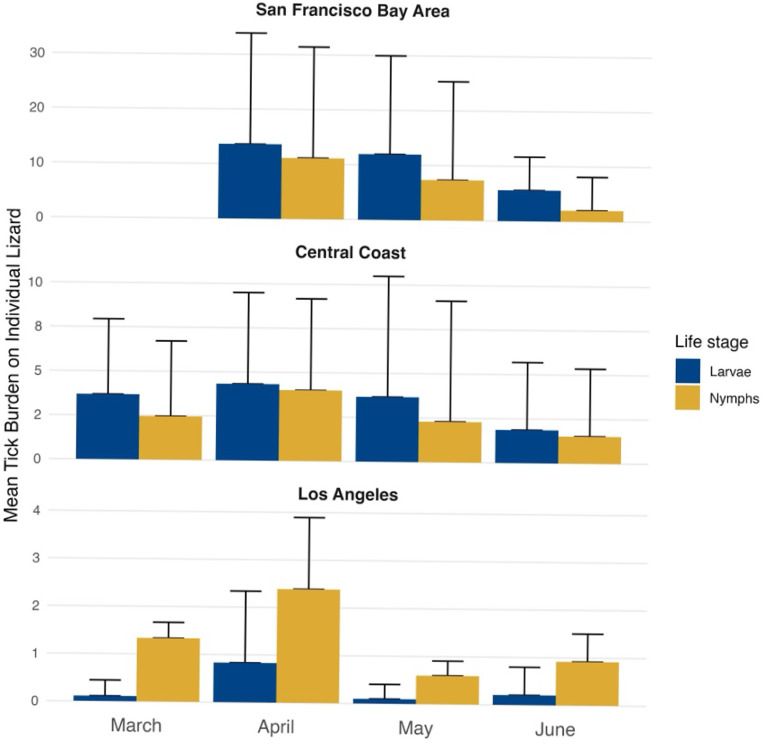
Mean tick burden across lizards per month, broken down by life stage and climate region. Upper error bars represent the standard error of the mean.

Tick abundances on lizards exhibited considerable variability across our sampling locations revealing several notable trends. The mean tick abundances on lizards ranged from 0.17 to 47.21 across locations and latitudinally declined with the San Francisco Bay Area exhibiting the highest tick burden (18.40 ± 23.07), followed by Central Coast (3.84 ± 6.62), and Los Angeles region (1.64 ± 3.99). Key regional patterns included: (i) San Francisco Bay Area had the highest mean tick burden across all locations (18 ticks per lizard); (ii) the Los Angeles region had the greatest proportion of lizards with zero ticks (> 50% of sampled lizards); and (iii) peak mean nymphal densities occurred 25 days earlier than peak larval densities in the San Francisco Bay Area only (Fig. 2). Additional results for juvenile ticks by climate region can be found in Additional file 4: Fig. S1 and Fig. S2.

##### Phenological dates

The Julian date of peak abundance – when the maximum mean burden per location was reached – for both larvae and nymphs on lizards was similar in the Central Coast and Los Angeles region (Julian date ~ 100 = April 9th). In contrast, the San Francisco Bay Area experienced peak abundance later in the season. Notably, nymphs in the San Francisco Bay Area reached their peak abundance earlier (Julian date 119 = April 29th) than larvae, whose peak abundance occurred later (Julian date 144 = May 24). Other notable phenological dates include the first Julian dates of appearance, which varied by region. The earliest tick appearance occurred in the Central Coast (Julian date 63 = March 4), followed by Los Angeles region (Julian date 83 = March 24), and then the San Francisco Bay Area (Julian date 94 = April 4).

##### Overlap metrics

Both Kernel Density Estimation (KDE) and the Jaccard Index (JI) revealed regional variation in the seasonal overlap between larval and nymphal populations on lizards. The San Francisco Bay Area exhibited the highest overlap (KDE = 0.89, JI = 0.47), followed by the Central Coast (KDE = 0.32, JI = 0.38), and the Los Angeles region (KDE = 0.30, JI = 0.22).

### Climate-associated patterns of juvenile tick abundances

**Figure 3:**
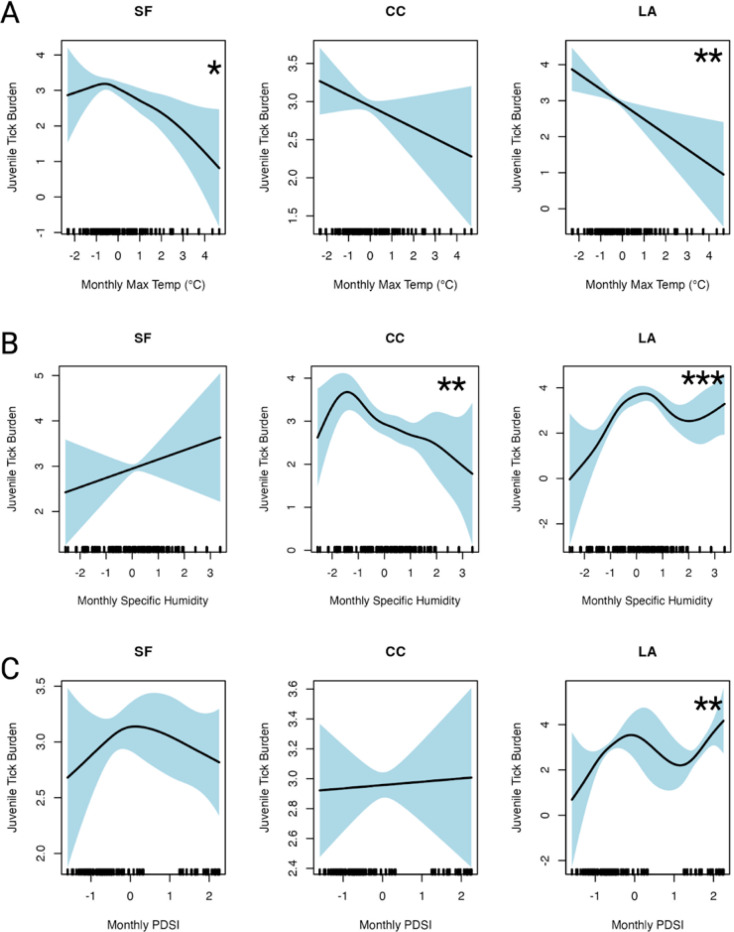
Smooth effect of climate predictors on total tick abundances across California from 2013–2022 from the Generalized Additive Model, stratified by climate region (SF = San Francisco, CC = Central Coast, LA = Los Angeles). Panels A, B, and C show the estimated relationship between tick abundances and monthly climate predictors: (A) maximum temperature, (B) specific humidity, and (C) the Palmer Drought Severity Index (PDSI - higher values, less drought). For each panel, the x-axis represents the standardized values of the predictor variables, and the y-axis shows the estimated effect on tick abundances (on the log_10_ scale). Asterisks (*) denote the significance level of the climate variable interaction switch climate region (p-value < 0.001***, < 0.01**, <0.1*).

To investigate how juvenile tick abundances are influenced by climate, we fit Generalized Additive Models to our juvenile abundances on individual lizards per sampling event (location-month-year). Our model for total tick abundances did not show significant linear relationships with climate regions, but it revealed important interactions between climate regions and abiotic variables through the smoothing terms (Fig. 3). Notably, the interaction with monthly maximum temperature showed a significant negative relationship with tick abundances: in the San Francisco region, tick abundances decreased as temperature rose above the regional mean (p = 0.02), while in Los Angeles, tick abundances were negatively associated with temperature but followed a stronger, linear pattern (p = 0.004). For San Francisco, the relationship was slightly nonlinear (edf = 3.0), with a sharp increase in tick abundances below the mean temperature, followed by a gradual decline above the mean. In contrast, the Los Angeles region exhibited a strongly linear relationship (edf = 1.0) where higher maximum temperatures led to lower tick abundances.

In addition, we found a significant positive effect of monthly specific humidity on tick abundances in the Los Angeles region (p < 0.001), whereas the Central Coast region showed an opposite trend, with tick abundances declining as humidity exceeded the regional mean (p = 0.003). Furthermore, the relationship between drought conditions (measured by the PDSI) and tick abundances was generally positive, with less drought (higher PDSI values) associated with greater tick abundances. However, this relationship was only statistically significant in Los Angeles (p = 0.002). Our total tick abundances model also accounted for spatial autocorrelation using a smoothing term for geographic location, which was highly significant (p < 0.001). Overall, the model explained 56.4% of the deviance, with an adjusted R^2^ of 47.2%. The model’s REML value was 3531.3, with a scale estimate of 3.38, and was based on 1,527 observations. Model diagnostics can be found in Additional file 5: Fig. S1.

Separate models for larval and nymphal abundances failed to converge when the spatial autocorrelation term (s(lon, lat, bs = “gp”)) was included but were successfully fitted with the other smoothing interaction terms. Results from these models are shown in Additional file 5: Fig. S2.

## Discussion

Our study reveals significant variation in juvenile tick phenology across California, suggesting that climate-driven changes in juvenile tick host-seeking behavior may contribute to regional differences in Lyme disease prevalence in ticks. By using lizards, the preferred host for juvenile *I. pacificus* ticks (25, 26), instead of tick drags in our investigation, we address previous challenges in detecting off-host juvenile ticks across the significant latitudinal gradient encompassing the distribution of this species. This study represents one of the most extensive spatial analyses of juvenile *I. pacificus* in California, providing valuable insights that could inform vector surveillance and improve our understanding of Lyme disease risk across a climatically diverse landscape.

We observed substantial variability in mean juvenile tick abundances on lizards, ranging from 0.17 to 47.21, with the highest mean abundances found in the San Francisco Bay Area and the lowest mean abundances in the Los Angeles region. Notably, in the San Francisco Bay Area, nymphs peaked 25 days earlier than larvae, a pattern not observed in the Central Coast and Los Angeles regions, where larvae and nymphs reached peak mean abundance on the same Julian date for each respective region. The earlier peak in nymphal abundances in the San Francisco Bay Area may increase the potential for Lyme disease transmission, as infectious nymphs could infect the rodent host population which will host the larvae that emerge later, amplifying infection rates (52). Such a timing difference in tick phenology may explain the higher Lyme disease prevalence in ticks observed in the San Francisco Bay Area, highlighting the importance of temporal dynamics in pathogen transmission (5, 31, 49, 53–55) (CDPH 2021). Some ecological hypotheses suggest that this pattern may emerge in the San Francisco Bay Area, rather than Central Coast or Los Angeles, due to the generally lower annual mean temperatures (22). These cooler conditions may allow juvenile ticks to extend their seasonal activity, enabling larvae to reach peak abundances later in the year compared to those in drier areas, where increased desiccation may prevent later peaks (56). Alternatively, juvenile abundances are higher in northern California than in southern California, which may enhance our ability to detect more pronounced differences in the seasonal activity of juvenile *I. pacificus* (22).

We also quantified the overlap between larval and nymphal populations and observed a clear latitudinal trend in this overlap. The highest overlap occurred in the San Francisco Bay Area, which was ~ 2 times higher than the Central Coast and ~ 3 times higher than the Los Angeles region. This finding contradicted our initial hypothesis that we would observe the highest juvenile overlap in warmer regions, such as Los Angeles, where a shorter season might intensify overlap due to an accelerated tick life cycle (56). However, this unexpected pattern may be influenced by the timing of our sampling in relation to tick emergence (Additional file 1:Table S2) and perhaps a longer season in northern than southern California (56). Yet prior studies in Mendocino, in northwestern California, suggest similar temporal patterns of juvenile overlap as our San Francisco Bay Area results (9, 57, 58). A more systematic sampling design, spanning pre- and post-season periods, would better capture these dynamics and provide a clearer picture of how overlap varies across regions. Finally, we found that in months with higher maximum temperature and with more severe drought conditions, tick abundances on lizards were lower (Fig. 3, Table 2). While these findings are consistent with previously documented relationships, we extend this understanding by demonstrating that both the shape and magnitude of these relationships vary across different climate regions (9, 19, 44, 59, 60).

Our results confirm that tick phenology patterns are latitudinally structured, with tick abundances highest in cooler northern regions compared to warmer southern regions (17, 56, 61). However, this simple north-to-south gradient narrative overlooks the complexity of tick seasonality, especially in a state with a large coastal climate influence that overlaps many established tick populations (38, 41, 62). Additional factors, such as humidity, which is influenced by proximity to the coast, can extend the tick season, as observed in the Central Coast region (Fig. 2) (19, 41). When examining the effect of humidity on total tick abundances, we found a strong positive relationship in the Los Angeles region, which is typically drier than Central California, suggesting that tick abundances in this region may be limited by moisture in the environment. In contrast, humidity above the regional mean decreased tick abundances in the Central California region, possibly because it surpassed the physiological tolerance threshold for juvenile ticks (19, 56). While we observed that tick activity starts earliest in the Central Coast region, followed by Los Angeles and finally San Francisco Bay Area, this temporal pattern may reflect sampling design rather than underlying ecological differences (Additional file 1: Table S2). An intriguing finding, though potentially influenced by sampling methods, was the earlier peak of nymphs compared to larvae in the San Francisco Bay Area. In California, it is generally assumed that larvae emerge at the same time as, or before nymphs, which may limit the transmission potential of the Lyme disease agent, as larvae can only acquire the bacterium through an infectious blood meal (63). In our study, however, nymphal abundances peaked 25 days prior to the larval peak. If this earlier peak in nymphs is accurate, it could indicate that more vertebrates are exposed to infectious blood meals before larvae emerge. However, we recognize that lizards do not directly provide infectious blood meals, but they may serve as proxies for broader juvenile tick activity patterns, potentially reflecting rodent tick burden dynamics (25–27).

While tick abundances on lizards may provide a more accurate method for estimating juvenile *I. pacificus* population dynamics compared to traditional drag cloth sampling, there are opportunities to refine this approach. Future studies should collect additional data on population density of lizards as well as characteristics of individual lizards, such as snout-to-vent length and sex, to better standardize results across different individuals, as these factors may influence total tick abundances (21, 24, 64). Our dataset included two primary species of lizards: alligator lizards and western fence lizards. While differences in size between these species could potentially introduce biases in tick averages, most alligator lizards were captured in southern California, where they exhibited, on average, lower tick burdens compared to their northern counterparts. Interestingly, these latitudinal trends in tick burdens closely mirrored those observed in western fence lizards. In the eastern US, variation in host-seeking behavior of *I. scapularis* ticks has been hypothesized to be driven by evolutionary differences in thermal tolerance between southern and northern populations. A similar mechanism may be influencing the behavior of *I. pacificus* populations, which could be explored through future molecular studies (14, 23). We acknowledge that repeated sampling at a single location, combined with data loggers (e.g., HOBO loggers), represents the gold standard for linking microclimatic conditions to tick seasonality. However, due to the time and logistical constraints associated with conducting such studies across multiple locations with diverse climate gradients, this approach may not be feasible for many research teams. We, therefore, recommend that future research incorporates unbiased data alongside the important metadata mentioned above, which will facilitate the synthesis of disparate ecological datasets to address broader ecological questions related to tick behavior and its implications for human health.

## Conclusions

This study provides new insights into the regional variation in juvenile *I. pacificus* phenology across California, revealing climate-driven differences in tick abundances that may help explain regional Lyme disease prevalence. Our findings suggest that factors such as temperature, coastal humidity, and drought severity influence tick seasonality, with notable differences in tick activity patterns across regions, particularly between the San Francisco Bay Area and Los Angeles regions. The earlier emergence of nymphs compared to larvae in the San Francisco Bay Area could increase the potential for Lyme disease transmission, which is consistent with variation in reported human incidence and epidemiological studies and warrants further investigation. By comparing data across a broad set latitudinal gradient, we offer valuable insights into juvenile tick dynamics and emphasize the need for continued longitudinal research to refine our understanding and inform vector control strategies.

## Supplementary Material

Supplement 1

## Figures and Tables

**Table 1:** Juvenile phenology metrics for three climate regions of California from 2013 to 2022. Density measures show the mean and standard deviations of tick abundances per region. Overlap metrics (kernel density estimation–KDE and Jaccard index) assess the degree of synchrony between larval and nymphal populations, with higher values indicating more overlap or similarity. Peak abundance refers to the Julian date when the mean abundance was highest in each region. For reference, Julian dates 101 and 144 correspond to April 11th and May 24th, respectively.

	Abundance		Overlap Metrics		Julian day of peak abundances	
	*Larvae*	*Nymphs*	*KDE*	*Jaccard*	*Larvae*	*Nymphs*
**San Francisco**	12.56 ± 19.1	9.39 ± 10.7	0.89	0.47	144	119
**Central Coast**	3.32 ± 5.5	2.56 ± 3.9	0.32	0.38	101	101
**Los Angeles**	0.32 ± 0.9	1.25 ± 1.7	0.30	0.22	104	104

**Table 2:** GAM models predicting log_10_-transformed juvenile tick abundance on individual lizards with climate variables standardized. Tweedie distribution with a log link function. Climate regions: San Francisco (SF), Central Coast (CC), Los Angeles (LA). Monthly climate variables: max. temperature (tmmx), specific humidity (sph), drought index (pdsi).

Outcome: total juvenile abundances per lizard					
Linear terms	Predictors	Estimates	SE	t-value	p-value
	SF (intercept)	2.72	5.79	0.47	0.64
	CC	−0.29	7.05	−0.041	0.97
	LA	−3.82	8.40	−0.46	0.69
Smooth terms		edf	Ref df	F	p-value
	tmmx - SF	3.084	3.76	2.93	0.018*
	tmmx - CC	1.0	1.00	2.044	0.15
	tmmx - LA	1.0	1.00	8.58	0.0034**
	sph - SF	1.0	1.00	0.76	0.38
	sph - CC	4.41	5.28	3.61	0.0025**
	sph - LA	4.27	5.013	5.94	< 0.001***
	pdsi - SF	2.17	2.71	1.56	0.29937
	pdsi - CC	1.00	1.00	0.033	0.86
	pdsi - LA	3.54	4.13	4.43	0.0023**
	Lon, Lat	26.00	27.59	14.21	< 0.001***

*** p-value < 0.001

** < 0.01

* < 0.1

Observations = 1,527

R^2^(adj) = 0.472

Deviance explained = 56.4%

REML = 3534.8; scale est 3.3677

## Abbreviations

CDD: cumulative degree days
CC: Central Coast region
GAMs: Generalized Additive Models
JI: Jaccard index or Jaccard similarity coefficient
KDE: kernel density estimation
LA: Los Angeles region
SE: standard error
SF: San Francisco Bay Area region

## References

[1] Rochlin I, Toledo A. Emerging tick-borne pathogens of public health importance: a mini-review. J Med Microbiol. 2020;69(6):781–91. 32478654 10.1099/jmm.0.001206 PMC7451033

[2] Diuk-Wasser MA, Gatewood AG, Cortinas MR, Yaremych-Hamer S, Tsao J, Kitron U, Spatiotemporal patterns of host-seeking Ixodes scapularis nymphs (Acari: Ixodidae) in the United States. J Med Entomol. 2006;43(2):166–76. 16619595 10.1603/0022-2585(2006)043[0166:spohis]2.0.co;2

[3] Ginsberg HS, Albert M, Acevedo L, Dyer MC, Arsnoe IM, Tsao JI, Environmental factors affecting survival of immature Ixodes scapularis and implications for geographical distribution of Lyme disease: the climate/behavior hypothesis. Pal U, editor. PLOS ONE. 2017;12(1):e0168723. 28076359 10.1371/journal.pone.0168723 PMC5226345

[4] Ostfeld RS, Brunner JL. Climate change and Ixodes tick-borne diseases of humans. Philos Trans R Soc B Biol Sci. 2015;370(1665):20140051. 10.1098/rstb.2014.0051 PMC4342967 25688022

[5] Kurtenbach K, Hanincová K, Tsao JI, Margos G, Fish D, Ogden NH. Fundamental processes in the evolutionary ecology of Lyme borreliosis. Nat Rev Microbiol. 2006;4(9):660–9. 16894341 10.1038/nrmicro1475

[6] Ogden NH, Mechai S, Margos G. Changing geographic ranges of ticks and tick-borne pathogens: drivers, mechanisms and consequences for pathogen diversity. Front Cell Infect Microbiol [Internet]. 2013 [cited 2022 Jun 13];3. Available from: http://journal.frontiersin.org/article/10.3389/fcimb.2013.00046/abstract 10.3389/fcimb.2013.00046 PMC3756306 24010124

[7] Hamer SA, Hickling GJ, Sidge JL, Walker ED, Tsao JI. Synchronous phenology of juvenile Ixodes scapularis, vertebrate host relationships, and associated patterns of Borrelia burgdorferi ribotypes in the midwestern United States. Ticks Tick-Borne Dis. 2012;3(2):65–74. 22297162 10.1016/j.ttbdis.2011.11.004

[8] Eisen L, Eisen RJ. Changes in the geographic distribution of the blacklegged tick, Ixodes scapularis, in the United States. Ticks Tick-Borne Dis. 2023;14(6):102233. 37494882 10.1016/j.ttbdis.2023.102233 PMC10862374

[9] Eisen RJ, Eisen L, Ogden NH, Beard CB. Linkages of weather and climate with Ixodes scapularis and Ixodes pacificus (acari: Ixodidae), enzootic transmission of Borrelia burgdorferi, and Lyme disease in North America. J Med Entomol. 2016;53(2):250–61. 26681789 10.1093/jme/tjv199 PMC4844560

[10] MacDonald AJ, McComb S, Sambado S. Linking Lyme disease ecology and epidemiology: reservoir host identity, not richness, determines tick infection and human disease in California. Environ Res Lett. 2022;17(11):114041.

[11] LoGiudice K, Ostfeld RS, Schmidt KA, Keesing F. The ecology of infectious disease: effects of host diversity and community composition on Lyme disease risk. Proc Natl Acad Sci. 2003;100(2):567–71. 12525705 10.1073/pnas.0233733100 PMC141036

[12] Allan BF, Keesing F, Ostfeld RS. Effect of forest fragmentation on Lyme disease risk. Conserv Biol. 2003;17(1):267–72.

[13] Gatewood AG, Liebman KA, Vourc’h G, Bunikis J, Hamer SA, Cortinas R, Climate and tick seasonality are predictors of Borrelia burgdorferi genotype distribution. Appl Environ Microbiol. 2009;75(8):2476–83. 19251900 10.1128/AEM.02633-08 PMC2675205

[14] Ginsberg HS, Hickling GJ, Burke RL, Ogden NH, Beati L, LeBrun RA, Why Lyme disease is common in the northern US, but rare in the south: the roles of host choice, host-seeking behavior, and tick density. Dobson AP, editor. PLOS Biol. 2021;19(1):e3001066. 33507921 10.1371/journal.pbio.3001066 PMC7842935

[15] Ogden NH, Beard CB, Ginsberg HS, Tsao JI. Possible effects of climate change on Ixodid ticks and the pathogens they transmit: predictions and observations. J Med Entomol. 2021;58(4):1536–45. 33112403 10.1093/jme/tjaa220 PMC9620468

[16] Eisen L. Climate change and tick-borne diseases: A research field in need of long-term empirical field studies. Int J Med Microbiol. 2008;298:12–8.

[17] Brinkerhoff R, Kitron U, Diuk-Wasser MA, Fish D, Melton F, Cislo P, Human risk of infection with Borrelia burgdorferi, the Lyme disease agent, in eastern United States. Am J Trop Med Hyg. 2012;86(2):320–7. 22302869 10.4269/ajtmh.2012.11-0395 PMC3269287

[18] Estrada-Peña A, Cevidanes A, Sprong H, Millán J. Pitfalls in tick and tick-borne pathogens research, some recommendations and a call for data sharing. Pathogens. 2021;10(6):712. 34200175 10.3390/pathogens10060712 PMC8229135

[19] Sambado S, MacDonald AJ, Swei A, Briggs CJ. Climate-associated variation in the within-season dynamics of juvenile ticks in California. Ecosphere. 2024;15(11):e70064.

[20] Copeland S, Sambado S, Orr D, Bui A, Swei A, Young H. Variable effects of wildlife and livestock on questing tick abundance across a topographical-climatic gradient. in press at Ecosphere. 2024;

[21] Schall JosJ, Prendeville HR, Hanley KA. Prevalence of the tick, Ixodes pacificus, on western fence lizards, Sceloporus occidentalis: trends by gender, size, season, site, and mite infestation. J Herpetol. 2000;34(1):160–3.

[22] Lane RS, Fedorova N, Kleinjan JE, Maxwell M. Eco-epidemiological factors contributing to the low risk of human exposure to Ixodid tick-borne borreliae in southern California, USA. Ticks Tick-Borne Dis. 2013;4(5):377–85. 23643357 10.1016/j.ttbdis.2013.02.005

[23] Arsnoe I, Tsao JI, Hickling GJ. Nymphal Ixodes scapularis questing behavior explains geographic variation in Lyme borreliosis risk in the eastern United States. Ticks Tick-Borne Dis. 2019;10(3):553–63. 30709659 10.1016/j.ttbdis.2019.01.001

[24] Ginsberg HS, Hickling GJ, Pang G, Tsao JI, Fitzgerald M, Ross B, Selective host attachment by Ixodes scapularis (Acari: Ixodidae): tick–lizard associations in the southeastern United States. Fonseca D, editor. J Med Entomol. 2022;59(1):267–72. 34718657 10.1093/jme/tjab181

[25] Casher L, Lane R, Barrett R, Eisen L. Relative importance of lizards and mammals as hosts for ixodid ticks in northern California. Exp Appl Acarol. 2002;26(1–2):127–43. 12475082 10.1023/a:1020911306291

[26] Slowik TJ, Lane RS. Feeding preferences of the immature stages of three western North American ixodid ticks (acari) for avian, reptilian, or rodent hosts. J Med Entomol. 2009;46(1):115–22. 19198525 10.1603/033.046.0115

[27] Eisen L, Eisen RJ, Lane RS. The roles of birds, lizards, and rodents as hosts for the western black-legged tick Ixodes pacificus. J Vector Ecol. 2004; 15709249

[28] Salkeld DJ, Lagana DM, Wachara J, Porter WT, Nieto NC. Examining prevalence and diversity of tick-borne pathogens in questing Ixodes pacificus ticks in California. Appl Environ Microbiol. 2021;87(13):e0031921. 33893109 10.1128/AEM.00319-21 PMC8316035

[29] MacDonald AJ. Abiotic and habitat drivers of tick vector abundance, diversity, phenology and human encounter risk in southern California. Munderloh UG, editor. PLOS ONE. 2018;13(7):e0201665. 30063752 10.1371/journal.pone.0201665 PMC6067749

[30] Salkeld DJ, Castro MB, Bonilla D, Kjemtrup A, Kramer VL, Lane RS, Seasonal activity patterns of the western black-legged tick, Ixodes pacificus, in relation to onset of human Lyme disease in northwestern California. Ticks Tick-Borne Dis. 2014;5(6):790–6. 25113980 10.1016/j.ttbdis.2014.05.002

[31] Padgett K, Bonilla D, Kjemtrup A, Vilcins IM, Yoshimizu MH, Hui L, Large scale spatial risk and comparative prevalence of Borrelia miyamotoi and Borrelia burgdorferi sensu lato in Ixodes pacificus. Stevenson B, editor. PLoS ONE. 2014;9(10):e110853. 25333277 10.1371/journal.pone.0110853 PMC4205013

[32] Wood SN. Generalized Additive Models: An Introduction with R [Internet]. 2nd ed. Chapman and Hall/CRC; 2017 [cited 2024 Nov 18]. Available from: https://www.taylorfrancis.com/books/9781498728348

[33] Lawrence A, O’Connor K, Haroutounian V, Swei A. Patterns of diversity along a habitat size gradient in a biodiversity hotspot. Ecosphere. 2018;9(4):e02183.

[34] Ackerly DD, Stock WD, Slingsby JA. Geography, climate, and biodiversity: the history and future of mediterranean-type ecosystems. In: Allsopp N, Colville JF, Verboom GA, editors. [Internet]. Oxford University Press; 2014 [cited 2022 Nov 15]. p. 361–76. Available from: https://academic.oup.com/book/6673/chapter/150719233

[35] Cal-Adapt. Climate regions for the 5th climate change assessment. Cal-Adapt website developed by University of California at Berkeley’s Geospatial Innovation Facility under contract with the California Energy Commission. Retrieved [22 August 2023], from https:://ucanr-igis.github.io/caladaotr/articles/api-requests.html; 2023.

[36] Swei A, Ostfeld RS, Lane RS, Briggs CJ. Impact of the experimental removal of lizards on Lyme disease risk. Proc Biol Sci. 2011;278(1720):2970–8. 21325326 10.1098/rspb.2010.2402 PMC3151702

[37] Abatzoglou JT. Development of gridded surface meteorological data for ecological applications and modelling. Int J Climatol. 2013;33(1):121–31.

[38] MacDonald AJ, McComb S, O’Neill C, Padgett KA, Larsen AE. Projected climate and land use change alter western blacklegged tick phenology, seasonal host-seeking suitability and human encounter risk in California. Glob Change Biol. 2020;26(10):5459–74. 10.1111/gcb.15269 32649017

[39] Bacon EA, Kopsco H, Gronemeyer P, Mateus-Pinilla N, Smith RL. Effects of climate on the variation in abundance of three tick species in Illinois. J Med Entomol. 2022;59(2):700–9. 34875079 10.1093/jme/tjab189 PMC8924963

[40] Ogden NH, Lindsay LR, Beauchamp G, Charron D, Maarouf A, O’Callaghan CJ, Investigation of relationships between temperature and developmental rates of tick Ixodes scapularis (Acari: Ixodidae) in the laboratory and field. J Med Entomol. 2004;41(4):622–33. 15311453 10.1603/0022-2585-41.4.622

[41] MacDonald AJ, O’Neill C, Yoshimizu MH, Padgett KA, Larsen AE. Tracking seasonal activity of the western blacklegged tick across California. Mosher B, editor. J Appl Ecol. 2019;56(11):2562–73.

[42] Varela L, Cooper M, Bentley W, Smith R. Degree-day accumulations used to time insecticide treatments to control 1st generation European grapevine moths. University of California Cooperative Extension [Internet]. 2011; Available from: https://www.aphis.usda.gov/sites/default/files/DDfor1stgenerationEGVM.pdf

[43] Levi T, Keesing F, Oggenfuss K, Ostfeld RS. Accelerated phenology of blacklegged ticks under climate warming. Philos Trans R Soc B Biol Sci. 2015;370(1665):20130556. 10.1098/rstb.2013.0556 PMC4342961 25688016

[44] Eisen RJ, Clark RJ, Monaghan AJ, Eisen L, Delorey MJ, Beard CB. Host-seeking phenology of Ixodes pacificus (Acari: Ixodidae) nymphs in northwestern California in relation to calendar week, woodland type, and weather conditions. J Med Entomol. 2017;54(1):125–31. 28082639 10.1093/jme/tjw155 PMC5229258

[45] Zuur A, Camphuysen C. Generalised Additive Models applied on northern gannets. In 2012. p. 145–68.

[46] R Core Team. R: A language and environment for statistical computing [Internet]. Vienna, Austria; Available from: https://www.R-project.org/

[47] Johnson M. climateR: climateR [Internet]. 2024. Available from: https://github.com/mikejohnson51/climateR

[48] Wickham H. ggplot2: Elegant Graphics for Data Analysis. 2nd ed. 2016. Cham: Springer International Publishing: Imprint: Springer; 2016. 1 p. (Use R!).

[49] Wickham H, Averick M, Bryan J, Chang W, McGowan L, François R, Welcome to the Tidyverse. J Open Source Softw. 2019;4(43):1686.

[50] Fox J, Weisberg S. An R Companion to applied regression [Internet]. Third edition. Thousand Oaks, CA: Sage Publications; 2019. Available from: https://socialsciences.mcmaster.ca/jfox/Books/Companion/

[51] Wright SA, Lane RS, Clover JR. Infestation of the southern Alligator lizard (Squamata: Anguidae) by Ixodes pacificus (Acari: Ixodidae) and its susceptibility to Borrelia burgdorferi. J Med Entomol. 1998;35(6):1044–9. 9835700 10.1093/jmedent/35.6.1044

[52] MacDonald H, Akçay E, Brisson D. The role of host phenology for parasite transmission. Theor Ecol. 2021;14(1):123–43. 34721722 10.1007/s12080-020-00484-5 PMC8549968

[53] Salkeld DJ, Nieto NC, Carbajales-Dale P, Carbajales-Dale M, Cinkovich SS, Lambin EF. Disease risk & landscape attributes of tick-borne Borrelia pathogens in the San Francisco Bay Area, California. Stevenson B, editor. PLOS ONE. 2015;10(8):e0134812. 26288371 10.1371/journal.pone.0134812 PMC4545583

[54] Fedorova N, Kleinjan JE, James D, Hui LT, Peeters H, Lane RS. Remarkable diversity of tick or mammalian-associated Borreliae in the metropolitan San Francisco Bay Area, California. Ticks Tick-Borne Dis. 2014;5(6):951–61. 25129859 10.1016/j.ttbdis.2014.07.015

[55] Shaw G, Lilly M, Mai V, Clark J, Summers S, Slater K, The roles of habitat isolation, landscape connectivity and host community in tick-borne pathogen ecology. R Soc Open Sci. 2024;11(11):240837. 39507992 10.1098/rsos.240837 PMC11540178

[56] MacDonald AJ, Briggs CJ. Truncated seasonal activity patterns of the western blacklegged tick (Ixodes pacificus) in central and southern California. Ticks Tick-Borne Dis. 2016;7(1):234–42. 26564403 10.1016/j.ttbdis.2015.10.016

[57] Eisen RJ, Eisen L, Lane RS. Predicting density of Ixodes pacificus nymphs in dense woodlands in Mendocino County, California, based on geographic information systems and remote sensing versus field-derived data. Am J Trop Med Hyg. 2006;74(4):632–40. 16606998

[58] Eisen RJ, Eisen L, Castro MB, Lane RS. Environmentally related variability in risk of exposure to Lyme disease spirochetes in northern California: effect of climatic conditions and habitat type. Environ Entomol. 2003;32(5):1010–8.

[59] McVicar M, Rivera I, Reyes JB, Gulia-Nuss M. Ecology of Ixodes pacificus ticks and associated pathogens in the western United States. Pathogens. 2022;11(1):89. 35056037 10.3390/pathogens11010089 PMC8780575

[60] Hacker GM, Jackson BT, Niemela M, Andrews ES, Danforth ME, Pakingan MJ, A comparison of questing substrates and environmental factors that influence nymphal Ixodes pacificus (acari: Ixodidae) abundance and seasonality in the Sierra Nevada Foothills of California. Fonseca D, editor. J Med Entomol. 2021;58(4):1880–90. 33860326 10.1093/jme/tjab037 PMC8529963

[61] Ogden NH, Pang G, Ginsberg HS, Hickling GJ, Burke RL, Beati L, Evidence for geographic variation in life-cycle processes affecting phenology of the Lyme disease vector Ixodes scapularis (Acari: Ixodidae) in the United States. J Med Entomol. 2018;55(6):1386–401. 29986046 10.1093/jme/tjy104

[62] Hahn MB, Feirer S, Monaghan AJ, Lane RS, Eisen RJ, Padgett KA, Modeling future climate suitability for the western blacklegged tick, Ixodes pacificus, in California with an emphasis on land access and ownership. Ticks Tick-Borne Dis. 2021;12(5):101789. 34280699 10.1016/j.ttbdis.2021.101789 PMC9379859

[63] Lane R S. Lyme disease in California: ecology and epidemiology. Proc Vertebr Pest Conf [Internet]. 2006 [cited 2022 Nov 14];22. Available from: https://escholarship.org/uc/item/2w83s251

[64] Dudek K, Skórka P, Sajkowska ZA, Ekner-Grzyb A, Dudek M, Tryjanowski P. Distribution pattern and number of ticks on lizards. Ticks Tick-Borne Dis. 2016;7(1):172–9. 26520053 10.1016/j.ttbdis.2015.10.014

