# Supplementary material for: Climate-driven variation in the phenology of juvenile Ixodes pacificus on lizard hosts
Source: Res Sq. 2024 Dec 23:rs.3.rs-5671938. Preprint. [Version 1] doi: 10.21203/rs.3.rs-5671938/v1 (PMC11703333; doi:10.21203/rs.3.rs-5671938/v1)
Supplement: Supplement 1 [file NIHPPRS5671938V1-supplement-1.pdf]

Supplementary Files

This is a list of supplementary files associated with this preprint. Click to download.

- ManuscriptLizardBurdenSupplemental20241218.docx
- GA.png
